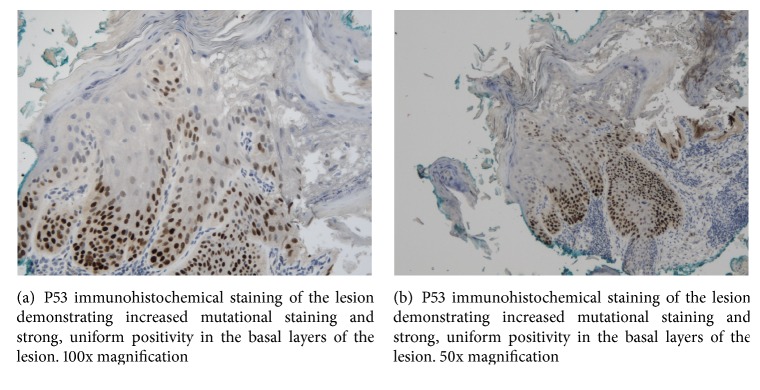# A Rare Presentation of Actinic Keratosis Affecting the Tarsal Conjunctiva and Review of the Literature

**DOI:** 10.1155/2018/4375354

**Published:** 2018-02-12

**Authors:** Selina Khan, Melanie Chak

**Affiliations:** Ophthalmology Department, Great Western Hospital, Swindon, UK

## Abstract

We report an unusual case of actinic keratosis (AK) of the tarsal conjunctiva in a 63-year-old man. Examination revealed a crusty, leukoplakic lesion prone to bleeding on the tarsal conjunctiva of the right upper eyelid. This was treated by surgical excisional biopsy. At 1-year follow-up, there was no evidence of recurrence and the surgical site was completely healed without conjunctival scarring. Current opinion cites excessive exposure to ultraviolet (UV) radiation, in particular UV-B in sunlight, as the causative agent in developing AK. In the case we present, the tarsal conjunctiva is an unusual place for actinic keratosis due to the lack of direct UV-light exposure. The key learning points are to evert the upper eyelid during examination especially if the lesion involves the eyelid margin and, secondly, to ensure risk factors are addressed during the history.

## 1. Introduction

Leukoplakia describes the whitish appearance of squamous epithelium after metaplastic keratinisation has occurred. Typically it affects nonkeratinised mucous membranes such as the oral cavity and larynx. There have been a few reports of leukoplakia of the conjunctiva and cornea. In the case we report, the leukoplakia was due to actinic keratosis (AK) also known as solar keratosis, of the tarsal conjunctiva and eyelid margin. The tarsal conjunctiva is an unusual presentation due to lack of direct UV radiation exposure.

## 2. Case Presentation

**Figure 1:**
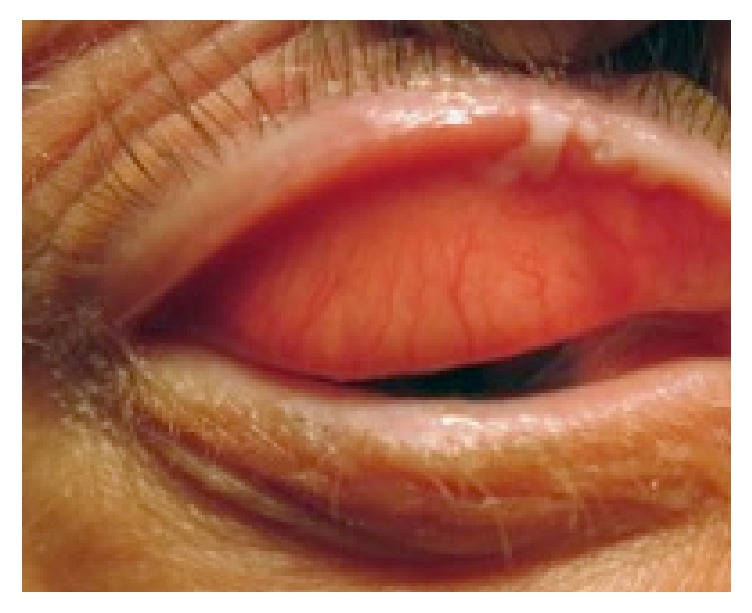
Photograph of the right-upper eyelid leukoplakia prior to excisional biopsy. Leukoplakia is present on eyelid margin and tarsal conjunctiva.

A 63-year-old, Caucasian man presented to outpatients' clinic with a crusty, bleeding, right upper eyelid lesion. This had gradually appeared over the past 12 months. Examination revealed two distinct lesions of leukoplakia on the right upper eyelid margin, extending onto the tarsal conjunctiva found on eye lid eversion and measuring 5 mm horizontally (Figure 1). Slit lamp biomicroscopy after 2% fluorescein staining showed multiple punctate epithelial erosions localised to the superior corneal surface. In 2013, he underwent surgical excision of a lesion to the right upper eyelid in a similar topography but limited to the skin. This was diagnosed as actinic keratosis on histological examination. He reported no significant history of prolonged sunlight exposure throughout his life. He had no history of skin cancer. He was not immunosuppressed.

**Figure 2:**
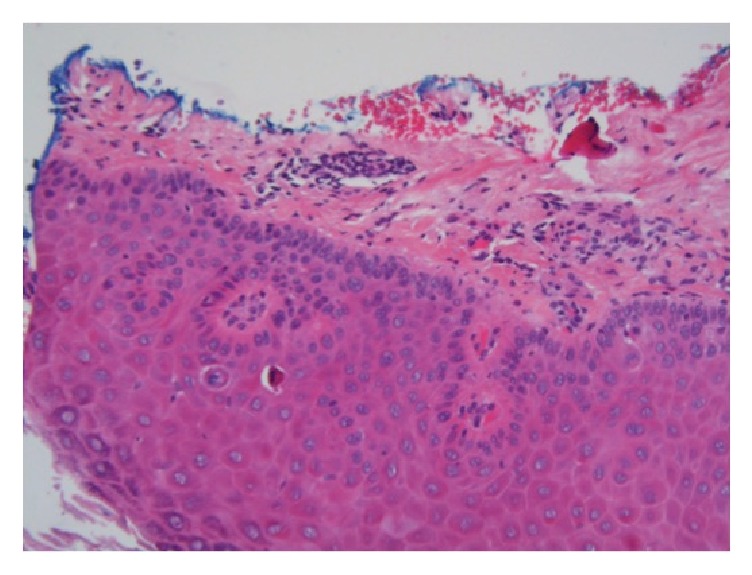
Histology specimen of the tarsal conjunctiva and eyelid margin. Marked hyperkeratosis and parakeratosis of the eyelid margin with extension of this cell atypia into the tarsal conjunctiva. Haematoxylin and Eosin, 100x magnification.

He was treated for the new lesions with a surgical excisional biopsy of the eyelid margin and tarsal conjunctiva under local anaesthetic. Histological examination confirmed features consistent with actinic keratosis on the eyelid margin and tarsal conjunctiva (Figures 2, 3(a), and 3(b)).

At 1-month follow-up after surgical excision, examination revealed complete resolution of the punctate epithelial erosions to the cornea and a healthy superior tarsal conjunctiva to both eyes. The cutaneous margin of the right eye, which was included in the biopsy, had fully healed without any complications. At 1 year follow-up, there was no evidence of recurrence.

## 3. Discussion

Actinic keratosis (AK) is defined as the neoplastic transformation and proliferation of keratinocytes within the epidermis without breaching the basement membrane [1]. Excessive exposure to UV radiation, primarily UV-B in sunlight, induces these changes which typically affect sun-exposed areas [2]. It is the chronic UV-B light exposure which results in damage to DNA and its repair mechanisms, specifically inducing mutations to the p53 gene, a key regulator of the cell cycle and tumourogenesis [3]. In the case of AK, transformation into squamous cell carcinoma follows the 3 stages of photocarcinogenesis: initiation, that is, UV-light exposure, promotion (clonal expansion of metaplastic cells), and conversion through further genetic mutations into squamous cell carcinoma [4].

In addition to cumulative sun exposure, other risk factors for AK include fair skin, advanced age, exposure to carcinogens, prolonged immunosuppression, and genetic conditions affecting DNA repair mechanisms such as xeroderma pigmentosum [5]. Most commonly AK is found on sun-exposed areas such as the hands, forearms, and face.

Clinically it is typically characterised by multiple, erythematous, nodular plaques ranging between 1 and 10 mm, unlike our case which presented with a leukoplakic lesion. The mainstay of cutaneous actinic keratosis treatment is complete excision biopsy. Alternatively topical chemotherapeutic agents such as 5-fluorouracil (5-FU) cream applied twice daily for 2-3 weeks and recently topical imiquimod 3 times per week for 16 weeks have been effective [1, 6, 7].

Reports of AK affecting the conjunctiva have been limited to the bulbar conjunctiva, with none reporting the management of tarsal involvement. These cases have been treated primarily with excisional biopsy and more recently with a combination of chemotherapeutic agents [8–11]. Rowlands et al. are the first group to successfully attempt complete eradication of bulbar conjunctival AK with 5% imiquimod after recurrence with cryotherapy and Interferon Alpha-2b [8]. Previous studies described no recurrence of actinic keratosis affecting the limbal conjunctiva when treated with excisional biopsy at 18 months' follow-up [9, 10].

Success with excisional biopsy for cutaneous AK is well-documented in the literature, less so but increasingly for bulbar conjunctival AK. Conservative therapy with topical agents with or without surgical excision remains an area to be further explored especially for multiple lesions.

A recent multicentre phase 2 trial reported complete clearance in 35.6% of individuals with AK to the face and chest with a once-daily for 3 days application regime of ingenol disoxate gel [12]. This is comparable to complete clearance rates for 5-FU, imiquimod, and diclofenac sodium 3% gel [13, 14]. Ingenol disoxate is a novel derivative to the ingenol mebutate molecule. This newer topical therapy induces rapid cell death and is able to exert its action in the dermis directly and indirectly by stimulating an immunogenic cytokine response. Consequently, it only requires a 2-3-day course of treatment which may increase compliance to treatment in comparison to alternatives such as 5-FU or imiquimod which require treatment for a minimum of 2 weeks. As of yet, there are no reports of its use in an ophthalmic context.

Treatment of periocular AK with topical diclofenac sodium 3% gel, used twice daily for up to 4 months, had resulted in complete resolution initially. However recurrence in 50% occurred in this study and further treatment with surgical excision was required in one case and adjuvant cryotherapy in another [15]. A case series of 13 patients with periocular AK treated with 5-FU for 14 days, twice a day, 9 of which with eye margin involvement, reported complete resolution at final follow-up. Five patients required a second course of 5-FU with minimal adverse reactions reported [6].

Our case report demonstrates successful, minimally destructive treatment of tarsal conjunctiva and eyelid margin AK with surgical excision without recurrence at 12 months. It also emphasises the importance of eyelid eversion for all patients with lesions involving the eyelid especially if adjacent to the margin. This is to check for the extension of the lesion and to allow for planning of appropriate management. Specifically, the tarsal conjunctiva rests adjacent to the cornea and when administering treatment to this region methods to minimise the risk of chemical keratopathy must be considered. It is also pertinent to ensure risk factors described above are asked during history-taking as susceptible individuals may need referral to a dermatologist if other suspicious lesions are identified.

Our case poses new questions on the exact pathophysiology of actinic keratosis of the tarsal conjunctiva. It specifically challenges the general understanding of the exact cause of AK and places the spotlight on the possibility of a contributory genetic component.

## 4. Conclusions

This is an unusual case of AK affecting a non-sun-exposed area and presenting with a leukoplakic appearance. Eversion of the upper eyelid is important to determine the extent of the lesion when it affects the eyelid margin, where possible excisional biopsy of AK is the first-line treatment of choice. New research into chemotaxic agents such as imiquimod has provided the opportunity for less invasive but effective treatment of conjunctival AK. In instances where complete excision is not viable, cryotherapy or imiquimod for conjunctival lesions is a second-line evidence-based therapy.

## Figures and Tables

**Figure 3:**